# Charting calcium-regulated apoptosis pathways using chemical biology: role of calmodulin kinase II

**DOI:** 10.1186/1472-6769-8-2

**Published:** 2008-08-01

**Authors:** Maria Hägg Olofsson, Aleksandra Mandic Havelka, Slavica Brnjic, Maria C Shoshan, Stig Linder

**Affiliations:** 1 From the Cancer Center Karolinska, Department of Oncology-Pathology, Karolinska Institute, S-171 76 Stockholm, Sweden

## Abstract

**Background:**

Intracellular free calcium ([Ca^2+^]_i_) is a key element in apoptotic signaling and a number of calcium-dependent apoptosis pathways have been described. We here used a chemical biology strategy to elucidate the relative importance of such different pathways.

**Results:**

A set of 40 agents ("bioprobes") that induce apoptosis was first identified by screening of a chemical library. Using p53, AP-1, NFAT and NF-κB reporter cell lines, these bioprobes were verified to induce different patterns of signaling. Experiments using the calcium chelator BAPTA-AM showed that Ca^2+ ^was involved in induction of apoptosis by the majority of the bioprobes and that Ca^2+ ^was in general required several hours into the apoptosis process. Further studies showed that the calmodulin pathway was an important mediator of the apoptotic response. Inhibition of calmodulin kinase II (CaMKII) resulted in more effective inhibition of apoptosis compared to inhibition of calpain, calcineurin/PP2B or DAP kinase. We used one of the bioprobes, the plant alkaloid helenalin, to study the role of CaMKII in apoptosis. Helenalin induced CaMKII, ASK1 and Jun-N-terminal kinase (JNK) activity, and inhibition of these kinases inhibited apoptosis.

**Conclusion:**

Our study shows that calcium signaling is generally not an early event during the apoptosis process and suggests that a CaMKII/ASK1 signaling mechanism is important for sustained JNK activation and apoptosis by some types of stimuli.

## Background

Calcium (Ca^2+^) is a universal signaling molecule regulating many aspects of cellular function and is one of the key elements of apoptotic signaling pathways [1]. Some agents, including glucocorticoids, the endoplasmic reticulum (ER) Ca^2+^-ATPase inhibitor thapsigargin and various cancer therapeutic drugs, mobilize Ca^2+ ^stores and trigger apoptosis by early transient elevation of intracellular free calcium ([Ca^2+^]_i_). However, elevation of [Ca^2+^]_i _has been described also at later stages of the apoptotic process [1]. A number of calcium-mediated apoptosis signaling mechanisms have been described. Mitochondria are located in microdomains close to Ca^2+ ^channels of the ER where cytosolic Ca^2+ ^concentrations may become high on channel opening [2]. Mitochondrial Ca^2+ ^uptake may result in mitochondrial permeability transition, leading to mitochondrial swelling and apoptosis. The calpains, calcium-activated cysteine proteases, have been implicated in apoptosis induction by some stimuli [3]. Calpain cleaves, and thereby activates, a number of molecules that have important functions in the apoptosis process, including caspase-12 [4], Bax [5] and Bid [6]. Calmodulin (CaM) is the major Ca^2+ ^sensor of nonmuscle cells [7] and signaling involving calmodulin has been implicated in apoposis [8]. Different signaling mechanisms down-stream from CaM are involved in various types of apoptotic responses, including pathways involving calcineurin, DAP kinase and calmodulin kinases. Calmodulin-dependent Kinase II (CaMKII) has been found to be both pro-apoptotic [9-12] and anti-apoptotic [13] in different studies.

The use of libraries of agents with diverse biological mechanisms allows for elucidation of new biological targets as well as mechanisms relevant to tumor cell viability [14]. Many previous studies have identified compounds that prevent the proliferation of tumor cells and several active compounds have been developed into clinically effective anticancer drugs. We here identified apoptosis-inducing agents in a compound library prescreened for agents that inhibit tumor cell proliferation. The objective of our study was to use the different agents as bioprobes to investigate the role of different calcium signaling mechanisms for apoptosis signaling. Apoptosis induction was analyzed in the presence and absence of a number of agents that influence Ca^2+ ^signaling. The results show that most, but not all, apoptotic drugs examined require calcium and that CaMKII is an important mediator of apoptotic signaling.

## Results

### Identification of apoptosis-inducing agents by screening

The aim of this study was to investigate the importance of different calcium pathways for induction of apoptosis of tumor cells. Since our study required a wide variety of apoptotic stimuli, we first set out to identify apoptosis-inducing agents by screening a chemical library. The NCI Mechanistic Drug Set, which contains 879 compounds selected from approximately 40,000 compounds based on different mechanisms of action with regard to cell growth inhibition, was screened to identify mechanistically diverse apoptosis-inducing agents. Apoptosis-inducing agents were identified using an assay which measures the accumulation of a caspase-cleavage product of cytokeratin-18 (CK18) in cultures containing dying and dead cells [15,16]; see Experimental procedures). Importantly, this assay provides an integrative measure of apoptosis and facilitates end-point measurements after treatment with collections of drugs that induce apoptosis with different kinetics. We used the human colon carcinoma cell line HCT116 as a screening target (p53wt; CK18+). Forty agents ("bioprobes") that induce strong caspase-cleavage activity were chosen based on representation of seven of the nine different response areas of the 3d MIND self-organizing maps [17]. Agents that segregate to the two remaining response areas (both with uncharacterized mechanisms) were not identified.

**Figure 1:**
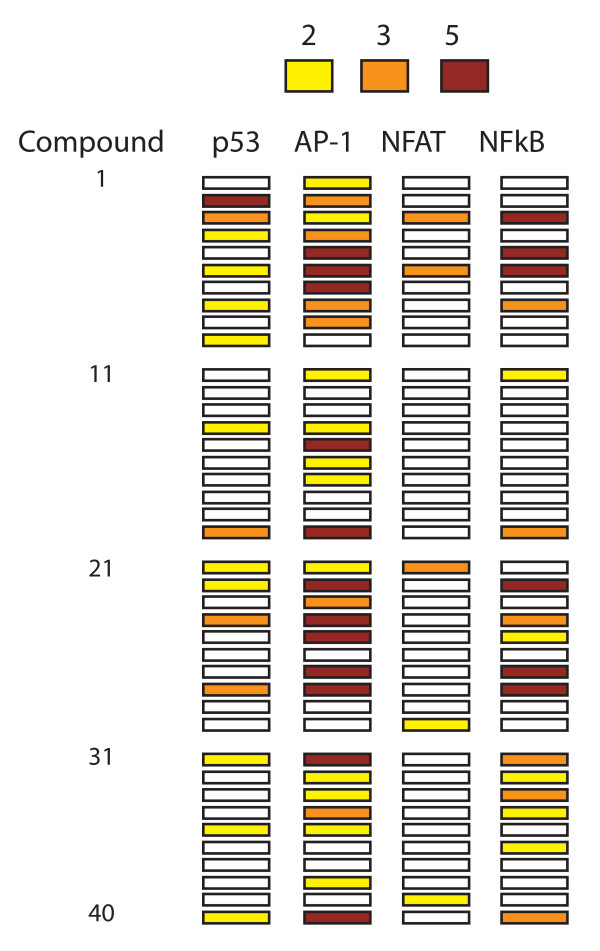
**Activation of reporter constructs by pro-apoptotic compounds**. Four different CellSensor^® ^HCT116 cell lines (Invitrogen) were treated with 40 different pro-apoptotic compounds and stimulation of reporter activity measured at 4 and 16 hours. Maximal stimulation at 4 or 16 hours time-point is illustrated graphically (yellow squares: >2-fold activation; orange squares: >3-fold activation; red squares: >5-fold activation).

The 40 bioprobes were used at the lowest possible concentration required for apoptosis induction. We first profiled the pattern of signaling induced by these bioprobes using four HCT116 cell lines which stably express different β-lactamase reporters for 4 pathways relevant to apoptosis signaling: p53, AP-1, NFAT and NF-κB (Fig. 1). AP-1 reporter induction was most frequently observed (30/40 compounds), whereas NFAT induction was most infrequent (5/40). A positive correlation was observed between induction of the AP-1 and NF-κB reporters (p = 0.012; Fishers exact test). We conclude that the bioprobes chosen on the basis of different mechanisms of growth inhibition also induce a diverse pattern of signaling.

### Examination of the role of calcium in apoptosis

**Figure 2:**
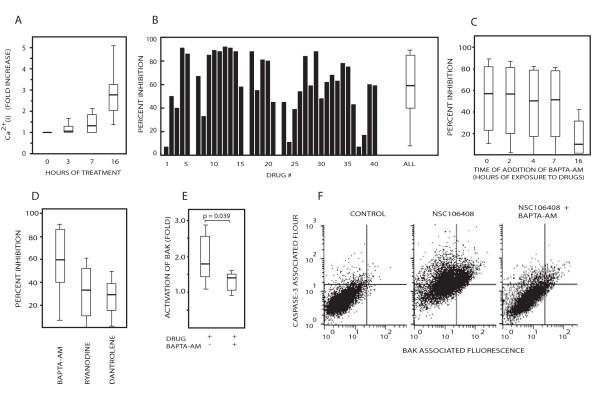
**Investigating the importance of calcium signaling pathways for apoptosis**. (A) Increases in Ca^2+^_(i) _evaluated by the fluorescent calcium indicator FLUO-4 after addition of the bioprobe set. Data in this and later figures are presented as box plots (median (25 – 75^th ^percentile; 10^th ^– 90^th ^percentile)); (B) HCT116 cells were treated with the bioprobe set for 24 hours in the presence or absence of 10 μM BAPTA-AM. The generation of the apoptosis-specific caspase-cleavage product CK18-Asp396 was assessed using the M30-Apoptosense^® ^ELISA assay [15] and the degree of inhibition of caspase-cleavage was calculated for each compound; (C) Ca^2+ ^is required during the late phase of apoptosis. BAPTA-AM was added at different times after addition of the bioprobe set and apoptosis was assessed at 24 hours of drug treatment; (D) Inhibiton of apoptosis by pre-treatment with ryanodine (10 μM) or dantrolene (50 μM), (E; F) BAPTA-AM blocks drug-induced modulation of the conformation of Bak. (E) HCT116 cells were exposed to 8 different drugs in the presence or absence of BAPTA-AM. All 8 drugs induced FLUO-4 fluorescence and BAPTA-AM sensitive apoptosis. Fixed cells were stained with an antibody that recognizes an N-terminal Bak epitope exposed after conformational activation during apoptosis [18,43]. (F) Inhibition of drug-induced caspase-3 activation and Bak conformation by BAPTA-AM. Cells were exposed to one of the bioprobes (NSC106408), fixed and stained by an anti-Bak antibody and an antibody to active caspase-3.

We used the FLUO-4 fluorescent calcium indicator to examine whether the bioprobes induced Ca^2+ ^fluxes in HCT116 cells. Twenty-seven of 40 (68%) bioprobes induced >50% increases in Ca^2+^_(i) _after 16 hours of exposure (median 2.7-fold increase). Smaller increases in Ca^2+^_(i) _were observed after 3 and 7 hours of drug exposure (Figure 2A). To examine the importance of calcium signaling for induction of apoptosis, cells were treated with the bioprobes in the presence or absence of the Ca^2+ ^chelator BAPTA-AM [1,2-bis(2-aminophenoxy) ethane-N,N,N1,N-tetra-acetic acid] followed by determination of caspase-cleaved CK18. BAPTA-AM inhibited the generation of caspase-cleaved CK18 by the majority of the bioprobes (Figure 2B). A median level of inhibition of approximately 60% was observed (Figure 2B, inhibition data for BAPTA-AM with regard to apoptosis induction by all bioprobes is presented as a box plot (median and spread)). Caspase-cleavage activity induced by some agents were not affected at all, showing that Ca^2+^_(i) _is not required for all types of apoptotic stimuli. Furthermore, induction of caspase-cleavage activity by thapsigargin (a SERCA pump inhibitor and inducer of ER stress) was inhibited by 85%.

BAPTA-AM could be added as late as 7 hours after drug addition and still provide significant protection from apoptosis (Figure 2C). Apoptosis was inhibited by blocking the ryanodine receptor (RyR) and inositol 1,4,5-triphosphate (InsP3) receptors using ryanodine and dantrolene, respectively (Fig. 2D). These results show that Ca^2+ ^is required for full induction of caspase-cleavage activity by many bioprobes but that Ca^2+ ^is not a mandatory requirement for all apoptotic signals. Results were validated by analysis of caspase-3 activity (for an example, see Figure 2F), loss of mitochondrial membrane potential (not shown) and activation of the conformation of the pro-apoptotic mitochondrial protein Bak (see below).

Many apoptotic signals involve the mitochondria, leading to release of molecules such as cytochrome c and AIF from mitochondria to the cytosol. The Bak and Bax proteins are essential regulators of mitochondrial permeabilization. Activation of Bak during apoptosis results in an altered molecular conformation, leading to altered conformation of the N-terminus of the protein [18]. BAPTA-AM was found to inhibit the conformational change of Bak after addition of 8 agents that showed calcium-dependent apoptosis induction (Figure 2E, F). We conclude from these experiments that calcium signaling is required at a late stage during apoptosis, but prior to conformational activation of Bak.

### Importance of calmodulin for drug-induced apoptosis

**Figure 3:**
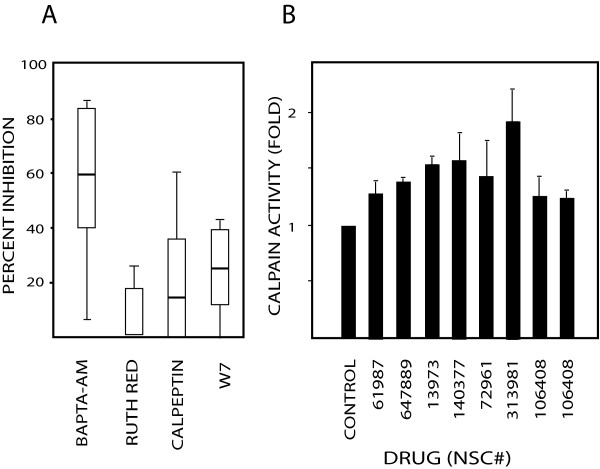
**Involvement of mitochondrial calcium uptake, calpain and calmodulin for apoptosis induction**. (A) Inhibition of generation of the caspase-cleaved CK18-Asp396 fragment by ruthenium red (5 μM), calpeptin (10 μM) or W7 (10 μM). HCT116 cells were individually treated with the 40 bioprobes in the presence inhibitors and CK18-Asp396 levels were determined by the M30-Apoptosense^® ^ELISA assay. Shown are median levels of inhibition for all 40 agents; (B) Induction of calpain activity of agents that showed calpeptin-sensitive apoptosis. Enzyme activity was measured after 18 hours using apoptosis-inducing drug concentrations.

We examined the relative roles of three processes known to be potentially involved in calcium-regulated apoptosis: mitochondrial calcium uptake, activation of the protease calpain and signaling involving calmodulin. Ruthenium red is a non-competitive inhibitor of the mitochondrial Ca^2+ ^uniporter [19], used to inhibit mitochondrial calcium uptake in cells [20,21]. Ruthenium red was found to inhibit apoptosis induced by some agents, but did not affect apoptosis induced by the majority of bioprobes (median level of inhibition: 0%; Figure 3A). Calpain is a Ca^2+^-activated protease involved in apoptosis signaling [5,6,22]. Calpeptin is a membrane-permeable inhibitor of both μ-calpain and m-calpain [3]. Calpeptin showed some inhibitory activity of apoptosis (Figure 3A), and increased calpain activity was accordingly detected after treatment with calpeptin-sensitive agents (Figure 3B). However, the median level of inhibition of caspase-cleaved CK18 was only one fourth of that observed using BAPTA-AM (15% versus 60%). We finally examined calmodulin, a central regulator of cellular Ca^2+ ^responses. We found that the calmodulin inhibitor W7 was more effective than calpeptin in inhibiting apoptosis, generating a median level of inhibition of 24% (Figure 3A).

### Relative importance of calmodulin-regulated pathways

**Figure 4:**
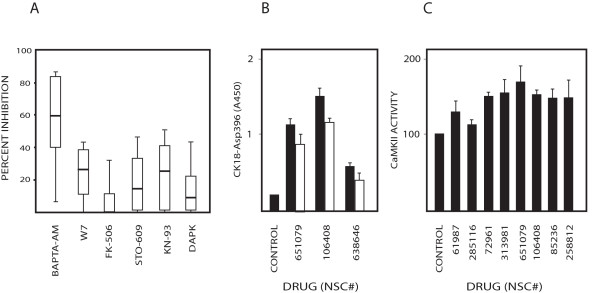
**Role of calmodulin signaling in apoptosis induction**. (A) Inhibition of caspase-cleavage activity by W7 (10 μM), the calcineurin inhibitor FK506 (10 μM), STO-609 (25 μM), KN-93 (10 μM) or DAPK siRNA. The efficiency of siRNA transfection on down-regulation was verified by western blotting. Inhibition of generation of CK18-Asp396 was calculated as in Fig. 3A; (B) Inhibition of caspase-cleavage activity by CaMKII siRNA. Cells were transfected with siRNA to CaMKII and exposed to the compounds indicated. CK18-Asp396 levels were determined by the M30-Apoptosense^® ^ELISA assay. Filled bars; control siRNA; open bars: CaMKII siRNA; (C) CaMKII enzymatic activity at 18 hours after treatment with selected compounds.

The finding that W7 inhibited apoptosis by several drugs prompted us to examine the potential involvement of different calmodulin-regulated pathways. Calcineurin (PP2B) is a Ca^2+^/calmodulin activated serine/threonine-specific phosphatase implicated in apoptotic signaling [23]. Calcineurin dephosphorylates NFAT, resulting in nuclear translocation and gene activation [24]. The antibiotic FK506 binds FKBP-12 and the resulting [FK506-FKBP-12] complex inhibits calcineurin activity [25]. FK506 did not significantly inhibit apoptosis induction by the bioprobe set (Figure 4A). We also examined the possible role of death-associated protein kinase (DAPK), a Ca2+/calmodulin-regulated protein kinase implicated in cell death [26]. Down-regulation of DAPK expression by siRNA did not significantly affect apoptosis induced by the bioprobe set (Fig. 4A), consistent with previous findings [27].

Other calmodulin-induced signaling pathways linked to apoptosis signaling involve calmodulin kinases. CAMKK-β was recently implicated in induction of macro-autophagy [28]. The CAMKK inhibitor STO-609 had a modest inhibitory effect on apoptosis (Figure 4A). CaMKII has been implicated in apoptosis by some stimuli [9,10,12]. Interestingly, the CaMKII inhibitor KN-93 reduced apoptosis to a similar degree as W7 (Figure 4A). Furthermore, treatment with CaMKII siRNA inhibited apoptosis induced by some examined compounds (Fig. 4B). Increases in CaMKII activity were observed after treatment by a number of compounds at 18 hours of treatment (Figure 4C). These results suggested that a pathway involving CaMKII is important in calcium-mediated apoptotic signaling down-stream of CaM.

### Role of CaMKII in apoptotic signaling

The experiments presented so far used the entire set of 40 bioprobes to examine to which extent different inhibitors affected apoptosis induction. This approach could not be used to specifically study a single pathway, since many of the compounds would be irrelevant. To specifically study CaMKII dependent apoptosis we chose the plant sesquiterpene lactone helenalin (NSC85236) which showed KN-93 sensitive apoptosis and that induced CaMKII activity. The selection was also supported by a previous report that helenalin causes increases in [Ca^2+^]_i _in 3T3 cells [29]. Importantly, in contrast to other candidates considered, helenalin has not been reported to induce reactive oxygen species or DNA damage. Others have, however, described helenalin as a specific inhibitor of NF-κB at 10 μM [30]. We here used helenalin at a concentration of 2 μM (see Discussion).

**Figure 5:**
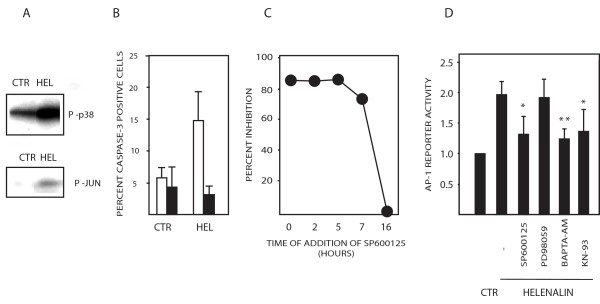
**Involvement of ASK-1 and JNK in helenalin-induced apoptosis**. (A) HCT116 cells were treated with 2 μM helenalin for 16 hours and ASK-1 activity (upper panel) or JNK activity (lower panel) was determined by immunokinase assays. (B) Cells were transfected with a dominant negative mutant of ASK-1 (black bars) or a control plasmid (open bars) and active caspase-3 was determined. The ASK-1 construct was HA-tagged and cells were stained with antibodies to HA and active caspase-3 followed by analysis by flow cytometry; (C) HCT116 cells were treated with 2 μM helenalin. SP600125 (5 μM) was added at the times indicated and apoptosis was assessed at 24 hours of drug treatment. The experiment was repeated with similar results. (D) Activation of an AP-1 reporter construct by helenalin. The CellSensor AP-1-*bla *HCT116 cell line (Invitrogen) was treated with 2 μM helenalin in the presence or absence of the indicated inhibitors. Activity was determined after 18 hours. Statistical significance is indicated relative to helenalin-only-treated cells (* p < 0.02; ** p < 0.002; Student's t-test).

Calcium signaling via CaMKII activates apoptosis signaling kinase 1 (ASK-1) [31,32]. ASK-1 is a serine/threonine protein kinase capable of activating the pro-apoptotic JNK pathway. HCT116 cells were treated with 2 μM helenalin and ASK-1 kinase activity was measured using an *in vitro *immunokinase assay with p38 as a substrate. Stimulation of ASK-1 activity was observed at 16 hours of treatment (Figure 5A; top panel). ASK-1 has been reported to be activated by calcium signaling [31,32], but there are few reports linking calcium signaling to JNK activation during apoptosis. We found that helenalin had induced JNK activity at 16 hours of treatment, assessed by an immunokinase assay (Figure 5A; bottom panel). In order to determine whether ASK-1 is required for helenalin-induced apoptosis, HCT116 cells were transfected with a dominant negative mutant of ASK-1. Strong inhibition of apoptosis was observed (Figure 5B). Furthermore, the JNK inhibitor SP600125 inhibited helenalin-induced apoptosis. Addition of the JNK inhibitor at 7 hours of drug treatment was sufficient for inhibition of apoptosis (Figure 5C). The observation of a late requirement for JNK is consistent with a late requirement for calcium and of an apoptosis-specific role for sustained JNK. Helenalin did not induce phosphorylation of p38 and apoptosis was not inhibited by the p38 kinase inhibitor SB203580 (not shown; etoposide was used as a positive control).

The levels of ASK-1 and JNK kinase activation by helenalin were moderate and the precision of the *in vitro *kinase assays was not found sufficient to reliably determine whether these kinases were inhibited by BAPTA-AM and KN-93. We therefore used the AP-1 reporter HCT116 cell line described in Fig. 1. Stimulation with phorbol ester and thapsigargin induced a reproducible increase in reporter activity (6.9-fold; C.V.: 17%). Helenalin induced a reproducible 2-fold induction of the AP-1 reporter at 18 hours of treatment (Fig. 5D). Reporter activity represents JNK activity since stimulation was decreased by the JNK inhibitor SP600125, but not by the ERK inhibitor PD98059. Using this assay we found that both BAPTA-AM and KN-93 inhibited helenalin-induced activation of the AP-1 reporter (Fig. 5D), showing that helenalin-induced AP-1 activity is dependent on calcium signaling.

## Discussion

Single-drug studies are limited with regard to the information about the relative contribution of various pathways to apoptosis. We here used a collection of 40 small molecular agents to induce apoptosis. These agents were selected from a larger drug set compiled on the basis of different mechanisms of growth inhibition of the NCI 60 cell line panel. Studies using a set of reporter cell lines confirmed that these compounds induced different patterns of signaling. This approach allowed a comprehensive evaluation of the role of calcium signaling in apoptosis. The data show that for many types of stimuli, calcium is generally required late during apoptosis induction. Calcium was likely to be released from intracellular stores, as evidenced by our finding that apoptosis was inhibited by blocking the ryanodine receptor (RyR) and the inositol 1,4,5-triphosphate (InsP3) receptor.

Of the calcium-regulated mechanisms examined, calmodulin-regulated pathways appeared to be most important for induction of apoptosis. Calmodulin has previously been implicated in apoptosis, both as a pro-survival [33] and as a pro-apoptotic factor [8]. CaMKII is a major mediator of cellular Ca^2+ ^effects and inhibition of this kinase was here found to inhibit apoptosis to a greater extent than inhibition of other candidates such as calpain and calcineurin/PP2B. The biological actions of CaMKII have been studied in most detail in intermediate metabolism and neural signaling [34]. CaMKII has been reported to be activated downstream of protease activation in apoptosis induced by UV light and TNF [9]. Induction of apoptotic cell death in hepatocytes by the toxin microcystin can be blocked by CaMKII inhibitors [10]. Furthermore, cadmium induces CaMKII and cadmium-induced apoptosis is dependent on this kinase [12]. Our data support these previous studies and suggest a common role for CaMKII in apoptosis by a number of different stimuli.

Other calmodulin-regulated pathways that were considered involved calcineurin and death-associated protein kinase (DAPK). Calcineurin is a widely expressed protein phosphatase regulated by Ca^2+^/calmodulin. Calcineurin dephosphorylates the NFAT transcription factor, resulting in nuclear translocation and gene activation [24]. Overexpression of calcineurin leads to apoptosis in cells deprived of growth factors [35]. Calcineurin dephosphorylates the pro-apoptotic Bcl-2 family protein BAD, leading to translocation of BAD to mitochondria [36]. Calcineurin has been implicated in p53-mediated apoptosis of colon carcinoma cells as down-stream mediator of calcium released from intracellular stores through the generation of reactive oxygen species [37]. Calcineurin did not appear to an important role for apoptosis induced by the set of bioprobes studies here, and we only found weak induction of NFAT. DAPK is a Ca^2+^/calmodulin-regulated protein kinase that mediates cell death [26]. There is little evidence in the literature that DAPK signaling is important for drug- or radiation-induced apoptosis of tumor cells [27]. We did not find any significant effect of DAPK down-regulation by siRNA on apoptosis induced by the bioprobe set.

Sustained JNK activation is recognized as a hallmark of many apoptotic processes [38]. Sustained JNK activation has been demonstrated to be associated with repression of the JNK-phosphatase MKP1 [39] but is also believed to be due to increased de novo phosphorylation by uncharacterized signaling pathways [40]. It is known that the calcium ionophore ionomycin induces JNK activity [41], but a role of calcium signaling for sustained JNK activation is not generally accepted. It is conceivable that calcium signaling contributes to apoptosis-specific sustained JNK activation via CaMKII. Of the different inhibitors that we have tested for inhibition of apoptosis by the current bioprobe set, BAPTA-AM and the JNK inhibitor SP600125 have provided the strongest degree of protection (median inhibition approximately 60% by BAPTA-AM; median inhibition 30% by SP600125). Other agents that we have been tested are the superoxide scavenger Tiron and the p38 kinase inhibitor SB203580, both of which were found to be less effective (MHO and AMH, unpublished data). JNK activation and calcium signaling pathways are likely to be of fundamental importance for apoptosis induced by many signals. That calcium signaling may be important for JNK activation is therefore an attractive possibility, but has not been as extensively studied as for example JNK activation by reactive oxygen/nitrogen species [42].

Small molecules are powerful tools for charting signaling pathways. It is, however, important to bear in mind that small molecules rarely affect single targets. This complicates the interpretation of the mechanism(s) of action of e.g. anticancer drugs. In the present study, we used the plant sesquiterpene helenalin to study the role of CaMKII in apoptosis and found that helenalin induced CaMKII, ASK-1 and JNK and that inhibiton of these kinases inhibits apoptosis. Helenalin may, however, have other effects and has in fact previously been described as a specific inhibitor of NF-kB when used at 10 μM [30]. We here used helenalin at 2 μM and did not observe NF-κB inhibition at this concentration (not shown). In fact, we have earlier found that when used at 10 μM, the response to helenalin was found to shift from apoptosis to necrosis [16].

## Conclusion

A number of calcium-regulated apoptosis pathways have been identified in various studies. Using a chemical biology approach with high throughput potential we have here both demonstrated a strategy for pathway charting as well as demonstrated an important role for CaMKII in apoptosis signaling in a human carcinoma cell line. Our data raise the possibility that calcium signaling is involved in sustained JNK activation during apoptosis induced by different stimuli.

## Methods

### Materials

The Mechanistic Drug Set was kindly provided by the Developmental Therapeutics Program, the National Cancer Institute, Bethesda, MD. This library contains 879 substanses that inhibit cell proliferation and/or induce cell death. The following compounds were identified to induce apoptosis by screening and chosen for the present study: NSC number: 622627 (4.0 μM), 69187 (7.0 μM), 10010 (4.0 μM), 285116 (2.0 μM), 647889 (6.0 μM), 157389 (7.0 μM), 24819 (1.0 μM), 13973(7.0 μM), 140377 (1.0 μM), 72961 (4.0 μM), 313981 (2.0 μM), 651079 (1.0 μM), 106408 (0.3 μM), 267461 (0.5 μM), 24818 (0.1 μM), 85236 (2.0 μM), 102811 (2.0 μM), 18268 (0.5 μM), 89671 (2.0 μM), 219734 (10.0 μM), 337766 (7.0 μM), 638646 (5.0 μM), 651080 (7.0 μM), 44690 (7.0 μM), 78365 (5.0 μM), 102866 (0.5 μM), 104129(10.0 μM), 164914 (1.0 μM), 258812 (0.2 μM), 407806 (1.0 μM), 635448 (2.0 μM), 673622 (1.0 μM), 679524 (2.0 μM), 684480 (7.0 μM), 328587 (5.0 μM), 693632 (2.0 μM), 175634 (5.0 μM), 68093 (5.0 μM), 4857 (0.2 μM) and 30916 (1.0 μM). These agents segregate into 7 of 9 possible response categories when tested on the NCI 60 cell line panel (see [17]): mitosis (M): 6 agents; membrane function (N): 7 agents; nucleic acid metabolism (S): 9 agents; metabolic stress and cell survival (Q): 8 agents; kinases/phosphatases and oxidative stress (P): 7 agents; uncharacterized regions R, F, J, and V: 0, 2, 0 and 3 agents. Compounds were stored in 100% DMSO in 96-well plates and added to cells using an EpMotion robot system (Eppendorf Nordic, Horsholm, Denmark). A final concentration of 0.5% DMSO was achieved in the cell cultures; control cultures were treated with this concentration.

### Cell culture

HCT116 colon carcinoma (p53wt) were generously provided by Dr. Bert Vogelstein and were maintained in McCoy's 5A modified medium supplemented with 10% foetal calf serum, L-glutamate, penicillin and streptomycin at 37°C in 5% CO_2_. Tissue culture reagents were obtained from Gibco Cell Culture Products. Transfection was performed using Lipofectamine 2000 (Invitrogen, Carlsbad, Cal) and 2 μg dnASK-1 mutant. DAP kinase siRNA was obtained from Qiagen (Hilden, Germany) and down-regulation was examined by western blotting.

### Reporter cell lines

Stable reporter HCT116 cell lines (CellSensor^®^) were obtained from Invitrogen (Carlsbad, CA). β-lactamase activity was determined using GeneBLAzer^® ^substrates from Invitrogen. β-lactamase activity leads to emission of blue light from the coumarin at 447 nm which was recorded in a Tecan Infinite F200 reader.

### Assessment of apoptosis

The accumulation of caspase-cleaved cytokeratin-18 (CK18-Asp396) was determined by the M30-Apoptosense^® ^ELISA assay [15] (PEVIVA AB, Bromma, Sweden). Cells were seeded at 10^4^/well in 96-well plates the day before treatment and treated with drugs as indicated. At the end of the incubation period, NP40 was added to the tissue culture medium to 0.1% and 25 μl of the content of each well (including activity released to the medium from attached and floating cells and cell fragments) was assayed for caspase-cleaved CK18. Activation of caspase-3 was investigated using an antibody specific for the active form of the enzyme. Paraformaldehyde fixed cells (0.25%, 5 min) were washed three times with PBS and incubated for 60 min with a fluorescein isothiocyanate-conjugated antibody recognizing active caspase-3 (BD Biosciences Pharmingen, San Diego, Cal). The antibody was diluted 1:50 in PBS containing digitonin (100 μg/ml). After incubation, the cells were washed with PBS, and fluorescence was monitored using the FL1 channel of a FACScalibur flow cytometer (BD, Franklin Lakes, NJ).

Upon induction of apoptosis, the proapoptotic Bak protein undergoes a conformational change that exposes an otherwise inaccessible N-terminal epitope [18]. We used an antibody shown to specifically recognize this epitope (amino acids 1 to 52 of Bak; EMD Chemicals, La Jolla, Cal). The increases in accessibility of the epitope were monitored by flow cytometry as previously described [43]. Data are presented as fold increase in immunofluorescence from control levels.

### Measurement of Ca^2+^_(i)_-

Drug-treated cells were harvested with cell dissociation solution (Sigma Aldrich, St Louis, MO) and the suspended cells were then incubated with the Ca^2+ ^indicator FLUO-4 (Molecular Probes, Invitrogen) for 60 s. The intracellular Ca^2+ ^levels, seen as fluorescent signal, were then assessed by flow cytometry using the FL1 channel.

### Enzyme activity measurements

CaMKII activity was assayed in cell extracts using the PROMEGA SignaTECT^® ^Assay which utilizes a specific biotinylated peptide substrate. Cells were harvested, washed with PBS and resuspended in Extraction buffer (50 mM HEPES pH 7.5, 5 mM EDTA, 1 mM Na-orthovanadate, 1 mM PMSF, Protease Coctail (1:50, Sigma Aldrich). Cells were lysed by four cycles of freeze-thawing in liquid nitrogen. Lysates were centrifuged (16,000 × g; 20 min) and supernatant fractions were collected. Enzymatic activity was assayed according to manufacturer's instructions. Calpain activity was assayed in cell extracts using the Calbiochem^® ^Calpain Activity Kit according to manufacturer's instructions. The data obtained were calculated as relative fluorescence units (RFU) and presented as fold untreated control.

### Immunokinase assays

The immunokinase assay for ASK-1 was performed as described [44]. In brief, immunoprecipitated ASK-1 was first incubated with GST-MKK6 (Jena Bioscience, Germany). The activated complex was then incubated with 0.3 mCi [γ-^32^P]ATP and 1 μg p38a^His ^(inactive; Jena Bio-science). After incubation for 10 min at room temperature, the reaction was terminated by addition of SDS-DTT and boiling. Thereafter samples were subjected to SDS-PAGE, followed by autoradiography. The immunokinase assay for JNK1-2 was performed after extraction in lysis buffer (RIPA buffer containing protease inhibitor cocktail (Sigma Aldrich), 1 mM PMSF, 2 mM DTT and 0.1 mM Na-orthovanadate). JNK1-2 were immunoprecipitated from cell extracts and the resulting immunocomplexes were bound to Protein A-Sepharose beads [45]. Beads were then resuspended in 20 mM HEPES pH 7.6, 2 mM DTT, 20 mM MgCl2, 1 mM PMSF, 0.1 mM Na-orthovanadate) containing 10 μM unlabeled ATP, 5 μCi, (γ-^32^P)ATP and 1 μg of recombinant GST-c-Jun and incubated at 30°C for 20 minutes. The reaction was terminated by addition of SDS-DTT and boiling. Thereafter samples were subjected to SDS-PAGE, followed by autoradiography.

### Statistics

Most of the data generated was not normally distributed and is presented as median (box plot; 25 – 75^th ^percentile; 10^th ^– 90^th ^percentile). The Wilcoxon two-sample test was used to compare levels between samples.

## Abbbreviations

CaM: Calmodulin; CaMKII: Calmodulin-dependent Kinase II ; RyR: Ryanodine receptor; InsP3: Inositol 1,4,5-triphosphate;  DAPK: Death-associated protein kinase; ASK-1: Apoptosis signaling kinase 1; TNF: Tumor necrosis factor;  NFAT: Nuclear factor of activated T-cells.

## Authors' contributions

MHO, AMH and SB performed the experimental work. MHC provided ideas and advice. SL provided ideas, funding and supervision for the work. All authors have read and approved the final manuscript.
